# Deletion of the GABA_A_ α2-subunit does not alter self administration of cocaine or reinstatement of cocaine seeking

**DOI:** 10.1007/s00213-014-3443-3

**Published:** 2014-01-31

**Authors:** C. I. Dixon, B. Halbout, S. L. King, D. N. Stephens

**Affiliations:** 1 School of Psychology, University of Sussex, Falmer, Brighton, BN1 9QG UK; 2 Present Address: Department of Microbiology and Molecular Genetics, University of California, Irvine, CA 92697 USA

**Keywords:** GABA, GABRA2, Addiction, Reinstatement, Cue

## Abstract

**Rationale:**

GABA_A_ receptors containing α2-subunits are highly represented in brain areas that are involved in motivation and reward, and have been associated with addiction to several drugs, including cocaine. We have shown previously that a deletion of the α2-subunit results in an absence of sensitisation to cocaine.

**Objective:**

We investigated the reinforcing properties of cocaine in GABA_A_ α2-subunit knockout (KO) mice using an intravenous self-administration procedure.

**Methods:**

α2-subunit wildtype (WT), heterozygous (HT) and KO mice were trained to lever press for a 30 % condensed milk solution. After implantation with a jugular catheter, mice were trained to lever press for cocaine (0.5 mg/kg/infusion) during ten daily sessions. Responding was extinguished and the mice tested for cue- and cocaine-primed reinstatement. Separate groups of mice were trained to respond for decreasing doses of cocaine (0.25, 0.125, 0.06 and 0.03 mg/kg).

**Results:**

No differences were found in acquisition of lever pressing for milk. All genotypes acquired self-administration of cocaine and did not differ in rates of self-administration, dose dependency or reinstatement. However, whilst WT and HT mice showed a dose-dependent increase in lever pressing during the cue presentation, KO mice did not.

**Conclusions:**

Despite a reported absence of sensitisation, motivation to obtain cocaine remains unchanged in KO and HT mice. Reinstatement of cocaine seeking by cocaine and cocaine-paired cues is also unaffected. We postulate that whilst not directly involved in reward perception, the α2-subunit may be involved in modulating the “energising” aspect of cocaine’s effects on reward-seeking.

## Introduction

GABA is the major inhibitory neurotransmitter in the mammalian brain but is often overlooked when considering the neurobiology of drug abuse. With particular reference to psychostimulant compounds, increases in synaptic dopamine in response to the drugs themselves and to drug-paired cues are well characterised (e.g., Broderick et al. 1993; Keller et al. 1992; Schultz et al. 1997; Volkow et al. 1999), as are alterations in glutamate signalling (Conrad et al. 2008; Thomas et al. 2001). However, within the nucleus accumbens (NAcc), a primary locus associated with integrating information relating to reward, 95 % of neurons are GABAergic medium spiny neurons (Taverna et al. 2004) with the remainder consisting mostly of GABAergic interneurons (Kawaguchi 1993). It is therefore important to consider how other reward-related neurotransmitter systems may interact with the inhibitory GABA neurons to mediate reward- and drug-related behaviour. Indeed, GABA-modulating compounds such as benzodiazepines and gammavinyl GABA, a GABA transaminase inhibitor, have been widely reported to decrease self-administration in the rat (Barrett et al. 2005; Filip et al. 2007; Kushner et al. 1999) and non-human primates (Negus et al. 2000; Weerts et al. 2005). Facilitation of GABAergic transmission also reduces the expression and acquisition of cocaine-conditioned place preference (CPP; Dewey et al. 1998) and behavioural sensitisation (Filip et al. 2006). Manipulations of inhibitory transmission have, therefore, been shown on many occasions to attenuate addiction- and reward-related behaviours and suggest that GABA may participate in the underlying neurobiology.

As the most prevalent GABA_A_ subunit within the NAcc (Pirker et al. 2000; Schwarzer et al. 2001), α2-subunit containing receptors are in a prime location to modulate information concerning reward and reward-related cues. In studies using the α2(H101R) mutant mouse strain, in which an amino acid substitution renders the α2-subunit insensitive to benzodiazepines (Low et al. 2000), diazepam is no longer able to decrease the reward threshold of intracranial self stimulation (Reynolds et al. 2012) or increase the locomotor stimulating properties of cocaine (Morris et al. 2008). These findings suggest that GABA acting at α2-subunit containing receptors contributes to both the perception of reward and the action of psychostimulant compounds. In addition to modulating reward pathways, specific activation of α2-subunit containing receptors, using Ro15-4513 in the α2(H101R) mutant mouse, induces behavioural sensitisation, whilst a deletion of the subunit resulted in a lack of sensitisation to cocaine (Dixon et al. 2010), suggesting activation of the α2-subunit is both necessary and sufficient for behavioural sensitisation to occur, and that these receptors thus potentially possess a direct role in reward-related behaviours.

Furthermore, human genetic studies have suggested that polymorphisms of the GABRA2 gene, encoding the GABA_A_ α2-subunit, are associated with dependence on many drugs of abuse, including ethanol (Covault et al. 2004; Edenberg et al. 2004; Enoch et al. 2006), heroin (Enoch et al. 2010), and nicotine (Agrawal et al. 2008), as well as polydrug use (Lind et al. 2008). It has also recently been noted that variations in this gene convey a risk for cocaine addiction (Dixon et al. 2010; Enoch et al. 2010). However, it is unclear how haplotypic variations translate to changes in expression or functionality of the receptor itself.

Considering the emerging evidence of a role for the GABA_A_ α2-subunit in modulating behaviours relating to psychostimulant reward, we tested whether motivation to obtain intravenous cocaine was altered in α2-subunit wildtype (WT), heterozygous (HT) and knockout (KO) mice. The propensity to reinstate drug seeking after extinction from repeated cocaine administration was also tested by presentation of a cocaine-related cue or a cocaine priming injection.

## Methods

### Animals

WT, HT and KO male mice were bred from heterozygous pairings and maintained on a mixed 50 % C57BL/6 J–50 % 129SvEv background. α2-Subunit KO mice were generated as previously described (Dixon et al. 2008). Animals were housed under a 12-h light/dark cycle (lights on at 7:00 a.m.) in a holding room with controlled temperature (≈21 °C) and humidity (≈50 %). Except where specified, animals had ad libitum access to standard laboratory chow (Bekay Feeds, Hull, UK) and water within the home cage. All experiments were carried out under the authority of the UK Animal (Scientific Procedures) Act, 1986.

### Drugs

Cocaine hydrochloride (MacFarlan Smith, Edinburgh, UK) was diluted in 0.9 % (*w*/*v*) sterile saline and administered i.p. at a volume of 10 ml/kg. For self-administration, cocaine was delivered intravenously in 0.02-ml infusions. The concentration of cocaine solutions was adjusted to achieve the required dose. For checking catheter patency, ketamine (Sigma-Aldrich, UK) and midazolam hydrochloride (Roche, UK) were dissolved in 0.9 % (*w*/*v*) sterile saline. A 0.02-ml intravenous infusion of combined ketamine (8.6 mg/ml) and midazolam HCl (0.42 mg/ml) was administered and catheter patency confirmed by a loss of righting reflex within 10 s of infusion.

### Apparatus

Mouse operant chambers (Med Associates, Vermont, USA) constructed of clear Perspex (18 × 18 × 15 cm), and contained in sound and light attenuating cubicles were used, containing two retractable levers. Stimulus lights were situated above each lever, with a food magazine between the levers and a speaker located above the magazine. The chambers were connected to a computer, which recorded behaviour using Med-PC Medical Associates software.

### Food training

WT (*n* = 18), HT (*n* = 25), and KO (*n* = 19) mice were food deprived to 85–90 % of free-feeding weight prior to starting the experiment. To minimise training time post-surgery and consequent loss of experimental animals, mice were trained to lever press on one lever (active lever) to receive a 100-μl aliquot of 30 % condensed milk solution, with the other lever (inactive lever) having no programmed consequence. Operant training started on an FR1 schedule (1 response required to obtain food delivery) for 20 reinforcers, increasing to FR2 for 10 reinforcers and an FR4 schedule for 10 reinforcers. The active lever was then switched and the cycle started from FR1 to avoid the animal developing a lever preference. After an initial 16-h session in the dark phase, mice were trained in daily 90-min sessions in the light phase until two lever cycles were completed on two consecutive days and 75 % of responses were performed on the active lever. Lever responses and sessions to criterion were recorded.

### Surgical procedure

Following food training, mice were returned to ad libitum chow access to allow their body weight to increase prior to surgery and remained free-feeding for the duration of the experiment. Mice were anaesthetised using a gaseous mix at 1 L/min flow rate (50 % N_2_O, 50 % O_2_) containing isofluorane (Abbott Laboratories, Maidenhead, UK). Anaesthesia was induced using 3 % isofluorane for approximately 3 min and maintained at 1–2 %, adjusted to keep the mouse in a surgical plane. Once anaesthetised, a sterile, platinum-cured silicon catheter (MIVSA 1050; CamCaths, Cambridgeshire, UK) was inserted into the jugular vein, secured with silk suture and passed subcutaneously to the centre of the back. The polypropylene mesh surrounding the external mounting of the catheter was secured under the skin using silk sutures. Catheters were flushed with 0.02-ml sterile saline containing 20 U/ml heparin on the first day and then daily with 0.02 ml containing 100 U/ml heparin after each self-administration session. For pain relief, mice were given 200 μl of a 1.5 mg/ml meloxicam solution (Metacam; Boehringer Ingelheim, Bracknell, UK) in mashed lab chow for 1 day prior and 3 days post-surgery. Mice were allowed to recover for 3 days before starting experiments.

### Cocaine self-administration

WT (*n* = 13), HT (*n* = 18), and KO (*n* = 13) were trained to lever press to receive a 0.02-ml infusion of 0.5 mg/kg cocaine on an FR4 schedule of reinforcement, with a maximum of 20 infusions to avoid potential negative consequences of overdose. Cocaine infusions were associated with a 10-s compound light and tone cue presentation (1 Hz flashing stimulus lights and a 2.9 kHz tone), during which all lever presses had no programmed outcome. After 10 sessions, catheter patency was confirmed using 0.02-ml intravenous infusion of midazolam HCl and ketamine solution resulting in a loss of righting reflex within 10 s. Mice without a viable catheter were excluded from the analysis and further experimentation.

### Reinstatement

Responding for cocaine was extinguished by placing animals (WT *n* = 9, HT *n* = 9, KO *n* = 11) in the operant boxes for 90 mins with lever pressing having no programmed consequence, until responding fell below 20 active lever presses per session. To test cue-induced reinstatement, mice were placed in the operant boxes for one 90-min session during which lever presses did not result in cocaine infusion, but resulted in a 10-s presentation of the previously cocaine-paired cue. Following cue-induced reinstatement, responding was extinguished again until active lever responses were below 20. To test for cocaine-induced reinstatement, mice were injected with 10 mg/kg cocaine i.p. and immediately placed in the operant boxes for 90 min, during which lever pressing had no programmed consequence. For all tests, active and inactive lever presses were recorded. Mice that failed to show any lever pressing behaviour were excluded from the experiment and analyses.

### Cocaine dose response

In a separate group of animals, after surgical implantation of an intravenous catheter, WT (*n* = 6), HT (*n* = 7), and KO (*n* = 6) were initially trained in daily 90-min sessions to lever press to receive a 0.02-ml infusion of 0.5 mg/kg cocaine on an FR4 schedule of reinforcement, paired with a 10-s compound cue, as described previously. On days 1 and 2, the maximum number of infusions was limited to 20 to avoid intoxication due to extinction from food training. After achieving the criterion for stable responding (>75 % responses on the active lever and <20 % variation in the number of infusions received over three consecutive days), the dose of cocaine was reduced daily according to the following: 0.25, 0.125, 0.06 and 0.03 mg/kg. At the end of the experiment, catheter patency was confirmed using a 0.02-ml intravenous infusion of midazolam HCl and ketamine solution. Mice without a viable catheter were excluded from the analysis.

### Cue-associated responding

To investigate cue-associated responding during maintenance of cocaine self-administration and the dose response study, the active lever presses occurring during cue presentation were taken into account. These presses occurred during the 10-s timeout period associated with the cue and, therefore, had no programmed consequence. For each session, the average number of active lever presses per number of cue presentations is presented.

### Statistical analyses

In the food training and dose response acquisition phases of the study, sessions to criterion were analysed using Kruskal-Wallis test. Genotype effects in reinstatement and extinction tests were analysed using one-way ANOVA. All other data were analysed using mixed-factor ANOVA. Dose response effects were analysed, following a significant ANOVA, with simple contrasts to the acquisition dose of 0.5 mg/kg.

## Results

### Food training

**Fig. 1:**
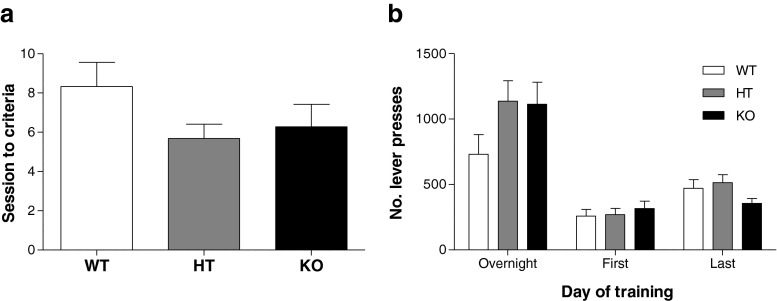
**a** Food training: sessions to criterion. WT, HT and KO learned the operant response to gain a condensed milk solution at a similar rate with no significant differences observed in the number of sessions to reach criterion. **b** Food training: active lever presses. Instrumental responding for the milk solution did not differ between genotypes, with all mice performing a comparable number of active lever presses in each training session

WT, HT and KO mice did not differ in their ability to learn an operant response to obtain a condensed milk reinforcer. All genotypes learned the association at a similar rate with no significant differences observed in the number of sessions to reach criterion (Fig. 1a; U_(2)_ = 3.222, *p* = 0.200). Instrumental responding for the milk solution also did not differ among genotypes, with all mice performing a comparable number of active lever presses in the initial overnight training session (Fig. 1b; F_(2,61)_ = 1.918, *p* = 0.156), the first daytime 90-min session (F_(2,61)_ = 0.327, *p* = 0.723) and the last training session once criterion was reached (F_(2,61)_ = 2.017, *p* = 0.142).

### Cocaine self-administration

**Fig. 2:**
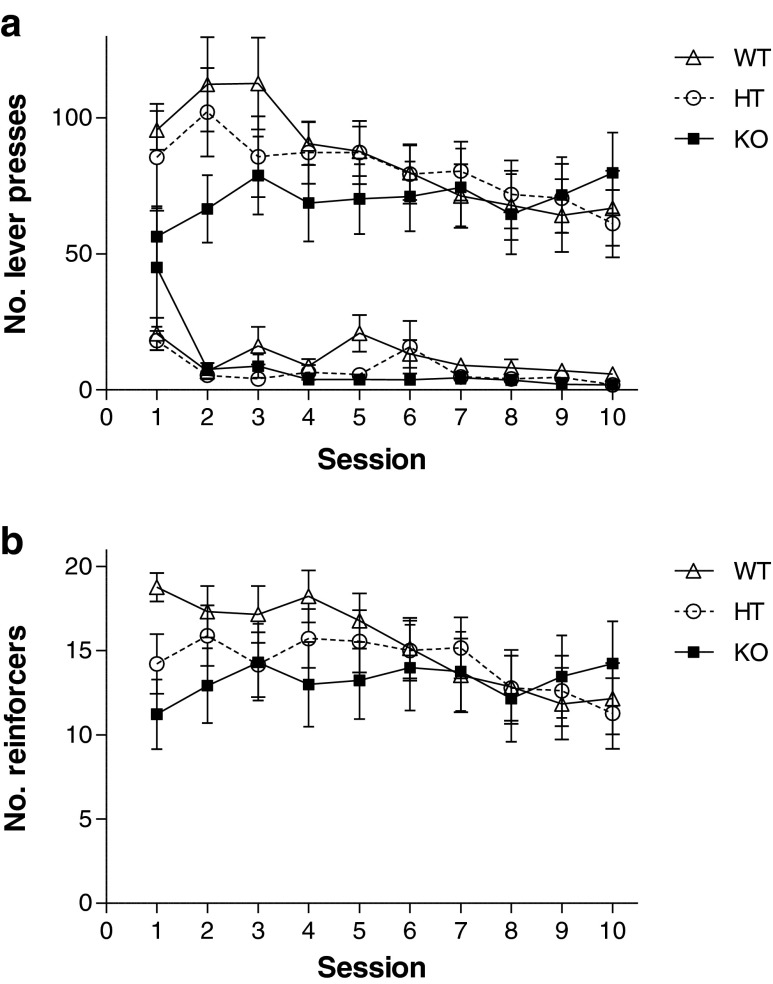
**a** Cocaine self-administration: lever presses. WT, HT and KO mice acquired self-administration of 0.5 mg/kg cocaine. The rate of active lever pressing was initially higher in WT and HT mice compared to KO but stabilised to a similar rate by session 4. **b** Cocaine self-administration: reinforcers. The number of cocaine infusions reduced over the course of the ten sessions in WT and HT mice but remained constant in the KO

All genotypes acquired self-administration of a 0.5 mg/kg cocaine infusion as indicated by a preference for the active lever (Fig. 2a; main effect of lever, F_(1,41)_ = 124.729, *p* < 0.001; non-significant lever by genotype interaction, F_(2,41)_ = 0.391, *p* = 0.679). However, the rate of lever pressing was initially higher in WT and HT mice compared to KO (lever by session by genotype interaction, F_(18,369)_ = 2.539, *p* < 0.01). Further analyses confirmed that whilst all genotypes reduced inactive lever responding during the course of the study (WT F_(9,108)_ = 2.168, *p* < 0.05; HT F_(9,153)_ = 2.424, *p* < 0.05; KO F_(9,108)_ = 3.401, *p* < 0.01), active lever responding reduced only in WT and HT mice, remaining consistent in KO animals (WT F_(9,108)_ = 3.989, *p* < 0.001; HT F_(9,153)_ = 2.561, *p* < 0.01; KO F_(9,108)_ = 1.341, *p* = 0.224).

During the ten sessions, the number of cocaine infusions received differed amongst genotypes (Fig. 2b; session by genotype interaction, F_(18,369)_ = 2.146, *p* < 0.01). As with the active lever presses, the number of infusions reduced over the course of the ten sessions in WT and HT mice (WT F_(9,108)_ = 4.977, *p* < 0.001; HT F_(9,153)_ = 2.265, *p* < 0.05) but remained constant in KO mice (F_(9,108)_ = 0.833, *p* > 0.587).

### Reinstatement

**Fig. 3:**
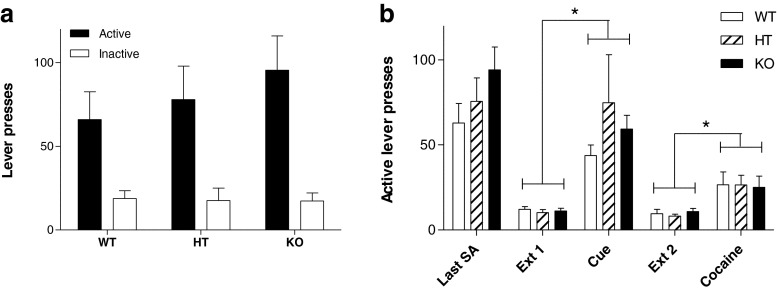
**a** First day of extinction. No significant differences in active or inactive lever presses were observed between genotypes when lever presses were no longer reinforced. **b** Reinstatement. No significant differences between genotypes were noted on the last day of cocaine self-administration or during either of the two extinction sessions. All three genotypes demonstrated a similar level of cue- and cocaine-induced reinstatement (* *p* < 0.05 compared to previous extinction session)

No significant differences between genotypes were noted on the last day of cocaine self-administration (Fig. 3b; F_(2,28)_ = 1.518, *p* = 0.238), the first day of extinction (Fig. 3a; F_(2,41)_ = 0.317, *p* = 0.730), or during either of the two extinction phases between reinstatement tests (Ext. 1: F_(2,28)_ = 0.331, *p* = 0.721; Ext. 2: F_(2,28)_ = 0.450, *p* = 0.643). Figure 3b shows that all three genotypes demonstrated a similar level of responding for the cocaine-associated cue (F_(2,28)_ = 0.843, *p* = 0.442) and during cocaine-induced reinstatement (F_(2,28)_ = 0.016, *p* = 0.984). Whilst no genotype effects were observed, when lever pressing resulted in cue presentation, lever pressing was significantly increased from extinction session 1 (F_(1,26)_ = 27.481, *p* < 0.001). A cocaine priming injection also significantly reinstated lever pressing on the active lever compared to the prior extinction test (F_(1,26)_ = 18.544, *p* < 0.001).

### Cocaine dose response

**Fig. 4:**
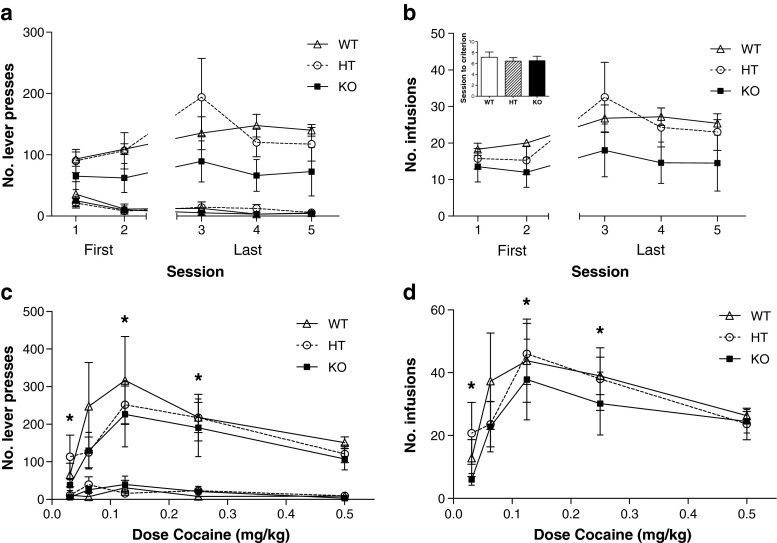
**a** Acquisition of cocaine self-administration: lever presses. No genotype differences were observed in the acquisition of self-administration for 0.5 mg/kg cocaine **b** Acquisition of cocaine self-administration: reinforcer. All three genotypes earned a similar number of reinforcers during acquisition of self-administration. WT, HT and KO mice learned the association at a similar rate, with no difference in the number of sessions to criterion **c** Cocaine dose response: lever presses. All mice-altered active lever responding in a dose-dependent manner (* *p* < 0.05 compared to training dose of 0.5 mg/kg cocaine). No genotype differences were evident **d** Cocaine dose response: reinforcers. WT, HT and KO mice also did not differ in the number of cocaine infusions received. However, the number of cocaine infusions received changed in a dose-dependent manner (* *p* < 0.05 compared to training dose of 0.5 mg/kg cocaine)

No genotype differences were observed in the acquisition of self-administration for 0.5 mg/kg cocaine. All genotypes showed similar lever pressing rates (Fig. 4a; lever by session by genotype interaction, F_(8,60)_ = 0.377, *p* = 0.929; main effect of genotype, F_(2,15)_ = 1.392, *p* = 0.279) and earned a similar number of cocaine infusions (Fig. 4b; session by genotype interaction, F_(8,60)_ = 0.266, *p* = 0.975; main effect of genotype, F_(2,15)_ = 1.100, *p* = 0.358). All genotypes also learned the association at a similar rate with no significant differences in the number of sessions to reach criterion for stable responding (Fig. 4b insert; U_(2)_ = 0.552, *p* = 0.759).

When allowed to self-administer decreasing doses of cocaine, mice-altered active lever responding in a dose-dependent manner (Fig. 4c; lever by dose interaction, F_(4,64)_ = 8.129, *p* < 0.001; main effect of dose on active lever, F_(4,64)_ = 8.649, *p* < 0.001). Simple contrasts to the training dose of 0.5 mg/kg revealed a dose-dependent increase in response at 0.25 and 0.125 mg/kg (F_(1,16)_ = 10.436, *p* < 0.01, F_(1,16)_ = 9.825, *p* < 0.01, respectively) and a decrease at 0.03 mg/kg (F_(1,16)_ = 4.571, *p* < 0.05). Lever responding at all doses did not differ among genotypes (lever by dose by genotype interaction, F_(8,64)_ = 0.936, *p* = 0.493).

WT, HT and KO mice also did not differ in the number of cocaine infusions received (Fig. 4d; dose by genotype interaction, F_(8,64)_ = 0.572, *p* = 0.912). However, overall, the number of cocaine infusions received changed in a dose-dependent manner (main effect of dose, F_(4,64)_ = 10.012, *p* < 0.001) paralleling the number of active lever presses (when compared to 0.5 mg/kg: 0.25 mg/kg, F_(1,16)_ = 7.999, *p* < 0.05; 0.125 mg/kg, F_(1,16)_ = 8.750, *p* < 0.01; 0.06 mg/kg, F_(1,16)_ = 0.270, *p* = 0.611; 0.03 mg/kg, F_(1,16)_ = 7.608, *p* < 0.05).

### Cue-associated responding

**Fig. 5:**
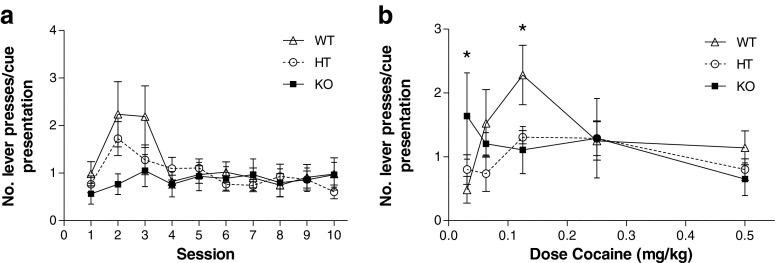
**a** Cue-associated responding: self-administration. Both WT and HT mice showed a change in cue-associated responding over the course of the ten sessions. KO mice remained consistent throughout all sessions with no change in active lever pressing. **b** Cue-associated responding: dose response. WT mice showed a dose-dependent increase in cue-associated responding (* WT: *p* < 0.05 from training dose of 0.5 mg/kg). HT and KO mice showed no significant change

Genotype differences were observed in the number of redundant lever presses made during the cocaine-associated cue presentation in both the initial single-dose cocaine self-administration study (Fig. 5a; dose by genotype interaction, F_(8,64)_ = 3.233, *p* < 0.01) and dose response studies (Fig. 5b; session by genotype interaction, F_(18,369)_ = 1.666, *p* < 0.05). Within the single-dose self-administration experiment, both WT and HT mice showed a change in cue-associated responding over the course of the ten sessions (F_(9,1.8)_ = 2.900, *p* < 0.01 and F_(9,153)_ = 3.869, *p* < 0.001, respectively). KO mice, however, remained consistent throughout all sessions with no change in active lever pressing (F_(9,108)_ = 0.519, *p* = 0.858). During the cocaine dose–response experiment, WT mice showed a dose-dependent increase in responding during cocaine infusion (and accompanying cue presentation) (F_(4,20)_ = 7.421, *p* < 0.01) with significant differences from the training dose at 0.125 mg/kg (F_(1,5)_ = 9.657, *p* < 0.05) and 0.03 mg/kg (F_(1,5)_ = 9.187, *p* < 0.05). HT and KO mice showed no significant change (F_(4,24)_ = 2.566, *p* = 0.064; F_(4,20)_ = 0.764, *p* = 0.328, respectively). Inactive lever presses have not been analysed as insufficient numbers were performed; on average, fewer than two presses on the inactive lever were performed during the cue presentations throughout the 90-min self-administration session.

## Discussion

The current experiments provide little evidence that GABA_A_ α2-subunit containing receptors are involved in cocaine taking as measured by intravenous self-administration. This conclusion is in agreement with a previous study that showed cocaine CPP to be unaffected in the α2-subunit knockout mouse (Dixon et al. 2010) and suggests that GABAergic transmission at GABA_A_ receptors containing α2-subunits is not importantly involved in signalling cocaine reward. We have also previously reported that deletion of the α2-subunit has no effect on self-administration of ethanol in an instrumental task (Dixon et al. 2012). In the present study, all genotypes learned the association between interoceptive and environmental cues associated with cocaine intake as assessed by the ability of such cues to reinstate drug-seeking behaviour after administration of either a cocaine priming injection, or as a consequence of access to the cocaine-associated cue being made contingent upon a lever press. Given the important role played by GABRA2 receptors in GABAergic inhibition of ventral striatal medium spiny neurons (Dixon et al. 2010) which play such an important role in drug reward, these findings are surprising.

Data from human studies have suggested that alterations in the GABRA2 gene encoding the α2-subunit influence liability for developing addictions to a variety of drugs, including alcohol and cocaine (Dixon et al. 2010; Edenberg et al. 2004; Enoch et al. 2006). Furthermore, mouse data from this laboratory have also shown an involvement of the α2-subunit in cocaine sensitisation (Dixon et al. 2010). Based on the hypothesis that cocaine sensitisation models the neuroadaptations in incentive systems underlying addiction (Robinson and Berridge 1993; Wise and Bozarth 1987) and our previous observations that behavioural sensitisation is absent in the knockout mouse, it might have been expected that a deletion of the α2-subunit would result in reduced incentive to take drug. Although some evidence was found during the first 2–3 sessions that α2-subunit KO mice showed lower rates of lever pressing, and obtained fewer cocaine infusions than WT mice, following additional training, all three genotypes showed similar patterns of responding, taking a similar number of infusions over the course of the 90-min session. The apparent initial lower rates of self-administration in the KO animals seems most likely to be attributable to an accelerated transition from prior food training to cocaine reinforcement, as indicated by increased responding by KO mice on the inactive lever in the first session of self-administration session (Fig. 2a), which was not evident in HT or WT mice. However, from session 3 onwards, mice from all three genotypes maintained responding at similar levels and there was no difference between genotypes in the dose–response curve. It thus seems appropriate to conclude that there was no marked reward deficit in the KO or HT mice. Alternatively, the decline in responding for cocaine over sessions in WT mice, while responding of KO mice remained stable, might reflect the development of sensitisation in WT mice, leading to a requirement for lesser drugs. The latter account would predict that the WT mice should show a shift to the left in the dose–response curve relative to KO mice, and experiment 2 specifically tested this possibility. Figure 4 illustrates that lever pressing and numbers of infusions were sensitive to dose, increasing at lower doses, while the lowest dose did not support self-administration. Nevertheless, although Fig. 4 suggests numerical differences between WT and KO mice, there were no significant dose by genotype interactions. These data thus do not support the notion that WT mice became more sensitive to the reinforcing properties of cocaine. Indeed, there was a non-significant tendency for WT mice to take more cocaine than KO mice in this experiment.

Although our findings are at one level surprising, they may be consistent with data from human studies that suggest that variations in the GABRA2 gene are linked to cocaine abuse only when in combination with early life traumatic events (Enoch et al. 2010). A similar consideration may apply to the apparent contradiction between our findings of no genetic differences in relapse models, and reports of increased risk of relapse associated with GABRA2 polymorphisms in humans (Bauer et al. 2012). However, the extent of reinstatement in rodent studies has been linked to the amount and schedule of prior drug exposure (Keiflin et al. 2008; Knackstedt and Kalivas 2007) and this is a factor that is difficult to control for in humans. Variations in the GABRA2 gene have been linked to conduct disorder (Dick et al. 2006), impulsivity (Villafuerte et al. 2012) and anxiety (Enoch et al. 2006), all of which are known to influence drug taking. The influence of the GABRA2 gene on drug taking and relapse in human subjects may, therefore, be due to interactions with these other psychological traits. In particular, a form of relapse which we have not been able to study in the present experiments is that induced by stress. Such a phenomenon is well established in humans (Fox et al. 2009; Sinha et al. 2006) and has also been demonstrated in rodent self-administration models (Ahmed and Koob 1997; Erb et al. 1996). Since α2-subunit containing GABA_A_ receptors have been strongly implicated in anxiety (Dixon et al. 2008), and the mechanism of action of anxiolytic agents (Dixon et al. 2008; Low et al. 2000; Morris et al. 2006) it seems possible that mice in which the α2-subunit has been deleted may show enhanced susceptibility to stress-related changes in drug behaviours.

How, then, to integrate our previously published findings that deletion of α2-subunit containing receptors prevents behavioural sensitisation to cocaine, and cocaine’s ability to facilitate responding for a conditioned reinforcer with the current data set showing little effect of the deletion on cocaine reward? The answer may lie in the several roles of dopamine release in ventral striatum in motivated behaviour. It is recognised that dopamine release may act both as a “teaching signal” (Schultz et al. 1997; Waelti et al. 2001), facilitating learning about environmental predictors of significant events, and to enhance the expression of behaviours directed to obtaining either primary or conditioned rewards/reinforcers (Herberg et al. 1976; Robbins and Everitt 2007; Salamone et al. 2005; Taylor and Robbins 1986). Thus, administration of cocaine increases food-conditioned activity (Le Merrer and Stephens 2006), increases expression of conditioned place preference (Maguire et al. 2014), and potentiates responding for conventional reinforcers such as sucrose under progressive ratio schedules (Brown and Stephens 2002; Thompson 1977) and for reward-related cues in conditioned reinforcement paradigms (Dixon et al. 2010). Whether this effect reflects the ability of cocaine to enhance the perception of reward, or to increase the salience of environmental stimuli, or simply to strengthen a motor output is yet to be elucidated. In fact, it is possible that these factors cannot be entirely separated and the outcome may be a culmination of some, if not all, of these effects. Whilst the action of psychostimulant compounds on conditioned reinforcement (CRf) is goal directed and peculiar to reward-paired stimuli (Katz 1976; Robbins 1978), the increase in operant responding also contains some perseverative behaviour and may encompass elements of stereotypy (Robbins 1976). However, the outcome remains to potentiate the expression of a cue-associated behaviour. It is this “energising” aspect of cocaine’s effects on reward-seeking that seems to be most consistently susceptible to manipulation of the GABA_A_ α2-subunit, while the consequences of facilitating dopamine transmission on reward or on the teaching signal may be less dependent upon modulation by GABA_A_ α2-subunit containing receptors.

Nevertheless, whilst measures of cocaine reward itself remain unaffected in the current experiments, a secondary finding emerged within these data that lever pressing during cue presentation following completion of the FR4 requirement to obtain cocaine, was absent in the KO mouse. Thus, during self-administration sessions, WT and HT mice, having successfully completed the FR4 response requirement to obtain an infusion of cocaine, continued to lever press during the 10-s light + tone cue presentation, though such behaviour had no consequences for obtaining further cocaine. As previously mentioned, response rates in the WT mice were higher at the beginning of the self-administration study and seem to be related to the transition from food to cocaine reward. Whilst this initial higher response rate could potentially confound the interpretation of redundant lever pressing, the extent of such responding depended on the cocaine dose in the dose response study, consistent with the behaviour being facilitated by cocaine rather than being simply a reflection of baseline response rate. Such an effect was not seen in either KO or HT mice. Furthermore, the dose response study was conducted at a later time point after food training than the self-administration study, at which point, the initial rate differences had been resolved. The genotype difference in redundant lever pressing rates does not appear to be a reflection of genotype on the rate-enhancing effect of cocaine since differences in response rates were not evident during FR4 responding within the self-administration dose response study or in the cocaine-primed reinstatement test. Additionally, it does not appear to be a secondary reinforcing effect of the cue alone since responding during the cue-induced reinstatement test did not differ amongst genotypes. We have reported previously that a deletion of the α2-subunit, whilst having no effect on acquisition of conditioned associations, or the expression of CRf for a food-related cue, impairs the ability of cocaine to increase such CRf responding (Dixon et al. 2010). We therefore speculate that the light + tone cue associated with cocaine delivery may acquire secondary reinforcing properties in both genotypes (as evidenced in the cue-induced reinstatement phase), so that lever pressing comes under the control of both cocaine- and cue-reinforcement, and this latter aspect is facilitated by the self-administered drug in WT mice only. When considered with our previously published findings, these data are consistent with our previous suggestion (Dixon et al. 2010) that when GABAergic inhibition via the α2-subunit is not present, increases in synaptic dopamine induced by cocaine administration can no longer potentiate cue-driven associations.

In conclusion, deletion of the GABA_A_ α2-subunit, whilst previously reported to abolish sensitisation to cocaine and cocaine-potentiation of conditioned reinforcement, does not affect self-administration of cocaine. Reinstatement of cocaine seeking by contingent presentation of a cocaine-paired cue or cocaine priming injection also remains intact. This is a surprising outcome considering the wealth of data within the human literature linking haplotypic variations of GABRA2 with various addictions, but suggests the role of GABA is more complex than simply mediating the perception of reward or reward-related stimuli.
